# Characteristics of Intestinal Microecology during Mesenchymal Stem Cell-Based Therapy for Mouse Acute Liver Injury

**DOI:** 10.1155/2019/2403793

**Published:** 2019-02-05

**Authors:** Xiaotian Dong, Xudong Feng, Jingqi Liu, Yanping Xu, Qiaoling Pan, Zongxin Ling, Jiong Yu, Jinfeng Yang, Lanjuan Li, Hongcui Cao

**Affiliations:** ^1^State Key Laboratory for the Diagnosis and Treatment of Infectious Diseases, First Affiliated Hospital, College of Medicine, Zhejiang University, 79 Qingchun Rd., Hangzhou City 310003, China; ^2^Collaborative Innovation Center for Diagnosis and Treatment of Infectious Diseases, 79 Qingchun Rd., Hangzhou City 310003, China

## Abstract

**Background:**

The mechanisms of mesenchymal stem cell (MSC) transplantation to protect against acute liver injury have been well studied within the liver. However, the associated changes in the intestinal microbiota during this process are poorly understood.

**Methods:**

In this study, compact bone-derived MSCs were injected into mice after carbon tetrachloride (CCl_4_) administration. Potential curative effect of MSC was evaluated by survival rate and biochemical and pathological results. Overall structural changes of microbial communities and alterations in the intestinal microbiota were assessed by sequenced 16S rRNA amplicon libraries from the contents of the cecum and colon.

**Results:**

MSCs significantly reduced the serum levels of aspartate transaminase and alanine transaminase and improved the histopathology and survival rate. Lower expression and discontinuous staining of zonula occludens, as well as disrupted tight junctions, were observed in CCl_4_-treated mice at 48 h compared with MSC-transplanted mice. Moreover, MSC transplantation to the liver leads to intestinal microbiota changes that were reflected in the decreased abundance of Bacteroidetes *S24-7* and *Bacteroidaceae* and increased abundance of Firmicutes *Clostridiales*, *Ruminococcaceae*, and *Lactobacillus* at the initial time point compared with that in CCl_4_-treated mice. In addition, phylogenetic investigation of communities by the reconstruction of unobserved states (PICRUSt) based on the Greengenes database revealed functional biomarkers of MSC-transplanted mice involved in cell motility, signal transduction, membrane transport, transcription, and metabolism of lipids, cofactors, vitamins, terpenoids, and polyketides, as well as xenobiotics.

**Conclusion:**

The initial alterations in the Firmicutes/Bacteroidetes ratio, which resulted from MSC infusion to the liver, maintain intestinal mucosal biology and homeostasis that may be beneficial to liver repair.

## 1. Introduction

The transplantation of mesenchymal stem cells (MSCs) demonstrates protective effects in various models of organ injury [1, 2], including carbon tetrachloride- (CCl_4_-) induced acute liver injury [3], implying that MSCs can be therapeutically effective [4–6]. However, the protective mechanisms have not been entirely defined. MSCs might protect against CCl_4_-induced acute liver injury by differentiating into hepatocyte-like cells [7], by an antioxidative process [8], or by paracrine secretions of cytokines, including interleukin-10 [3], and extracellular vesicles [9], including exosomes [10]. Because acute liver injury impairs the intestinal mucosa structure and tight junctions (TJs) [11, 12], resulting in bacterial translocation and portal endotoxemia that can serve as a contributory mechanism of hepatotoxicity [13–15], we considered that the therapeutic effect of MSCs might involve microbiota changes that promote barrier integrity.

Signals from the gut microbiota have been associated with maintenance of healthy host functions and various diseases, including nonalcoholic fatty liver disease/nonalcoholic steatohepatitis (NAFLD/NASH), type 1/2 diabetes, obesity, inflammatory bowel disease, and autism [16]. For liver diseases, microbial dysbiosis, exposing the gut mucosal cells to potentially harmful substances, including enteric bacterial pathogens [17], lipopolysaccharide (LPS), and endotoxins, as well as secreted cytokines (e.g., tumor necrosis factor-*α*) [18], can disrupt TJs and increase intestinal permeability and expose the liver to potentially proinflammatory bacterial products that may promote hepatic steatosis [19, 20]. However, the role of gut microbiota in MSC therapy of acute liver injury remains unknown. This is the first study to reveal the gut microbiome and its related pathways, as well as intestinal epithelial TJs, following MSC treatment of CCl_4_-induced acute liver injury.

## 2. Materials and Methods

### 2.1. Animals

Eight-week-old male wild-type C57BL/6 mice (weight, 20–25 g) (Shanghai SLAC Laboratory Animal Co. Ltd., Shanghai, China) were used for the induction of acute liver injury, and inbred 2-week-old green fluorescent protein (GFP) transgenic mice (male and female, C57BL/6 background, Nanjing Biomedical Research Institute of Nanjing University, Nanjing, China) were used for the isolation and culture of MSCs. Wild-type C57BL/6 mice were housed in a barrier environment, and GFP transgenic mice were housed in an insulated environment. All animals received humane care, and all procedures were approved by the Animal Care Ethics Committee of the First Affiliated Hospital, Zhejiang University.

### 2.2. Cells

The isolation and culture of mouse MSCs from compact bone were performed as previously described [21]. The cells were cultured in an MSC medium (OriCell™ C57BL/6 MSC complete medium; Cyagen Biosciences, Guangzhou, China) at 37°C in a 5% CO_2_ incubator (HERAcell®150; Thermo Fisher Scientific Inc., Waltham, MA, USA). MSCs from passages 5 and 7 were used throughout the experiments. The phenotype and multipotential differentiation of infused MSCs were investigated (Supplementary Figure S1 online).

### 2.3. CCl_4_-Induced Acute Liver Injury and Sample Collection

The solution of CCl_4_ (Sigma-Aldrich, St. Louis, MO, USA) and olive oil (Sigma-Aldrich) at a ratio of 1 : 1 and dose of 3 mL/kg body weight was administered intraperitoneally (i.p.) in a single dose. Control mice were administered an i.p. injection of an equal volume of olive oil (3 mL/kg) only. Next, 5 × 10^5^ MSCs in 100 *μ*L of phosphate-buffered saline (PBS) or 100 *μ*L of PBS were injected into the mice via the caudal vein 6 h after CCl_4_ administration.

The mice were anesthetized by i.p. injection of 4% chloral hydrate (Sangon Biotech, Shanghai, China) at a dose of 10 *μ*L/g body weight, and blood samples were drawn from the inferior vena cava at 48 h, 1 week (w), and 2 w after CCl_4_ administration. Following exsanguination, the liver, small intestinal segments, and cecum and colon contents were precisely dissected and harvested, snap frozen in liquid nitrogen, and stored at -80°C until further analysis. Portions of the liver and ileum were immediately fixed in 10% neutral buffered formalin for histopathologic examination.

### 2.4. Liver Biochemistry and Intestinal and Liver Histology

For histopathologic analysis, formalin-fixed liver and ileum samples were paraffin-embedded, sectioned (5 *μ*m thickness), and stained with hematoxylin and eosin. The sections were randomly numbered prior to reading and observed by an experienced pathologist. Images were acquired using a NanoZoomer 2.0-RS scanner (Hamamatsu Photonics KK, Hamamatsu City, Japan) equipped with scanner software. Terminal deoxynucleotidyl transferase-mediated dUTP nick end labeling (TUNEL) staining (Roche Diagnostics GmbH, Mannheim, Germany) was performed following the manufacturer's instruction and counterstained with 4′, 6-diamidino-2-phenylindole (DAPI). The alanine aminotransferase (ALT) and aspartate aminotransferase (AST) activities of serum samples were measured using a dry chemistry analyzer (FUJI DRI-CHEM 4000ie; Fujifilm Corporation, Tokyo, Japan) according to the manufacturer's instructions.

### 2.5. Real-Time Quantitative Polymerase Chain Reaction

Total RNA was isolated from ileum tissue using TRIzol reagent (Life Technologies Corporation, Carlsbad, CA, USA). cDNA was synthesized from 1 *μ*g of total RNA using the QuantiTect Reverse Transcription Kit (Qiagen, Hilden, Germany). Real-time quantitative polymerase chain reaction (qPCR) was performed using the ABI 7500 Real-Time PCR system and SYBR Premix Ex Taq™ II Kit (Takara Bio Inc., Shiga, Japan) according to the manufacturer's instructions. Oligonucleotide primers for mouse zonula occludens- (ZO-) 1 (forward 5′-ACTCCCACTTCCCCAAAAAC-3′ and reverse 5′-CCACAGCTGAAGGACTCACA-3′) and *β*-actin (forward 5′-ACAGGCATTGTGATGGACTC-3′ and reverse 5′-ATTTCCCTCTCAGCTGTGGT-3′) were synthesized by Sangon Biotech.

### 2.6. Immunofluorescence Analysis of ZO-1

Formalin-fixed, paraffin-embedded sections of the ileum were deparaffinized, heated in citrate buffer (pH 6.0; Wuhan Goodbio Technology Co. Ltd., Wuhan, China), and blocked with PBS containing 5% bovine serum albumin (Sangon Biotech Corp.) at room temperature for 30 min. The sections were incubated with primary rabbit anti-ZO-1 antibody (1 : 400; Invitrogen Life Technologies, Carlsbad, CA, USA) at 4°C overnight. Subsequently, the sections were incubated with Alexa488-conjugated goat anti-rabbit IgG (1 : 50; Invitrogen Life Technologies) at room temperature for 60 min, and the nuclei were counterstained with 4′,6-diamidino-2-phenylindole for 2 min. The sections were then examined using a confocal microscope system (Zeiss LSM-710; Carl Zeiss AG, Oberkochen, Germany). Images were acquired using ZEN 2012 software.

### 2.7. Transmission Electron Microscopy

The ileum samples were immediately fixed using 2.5% glutaraldehyde (Sangon Biotech) and kept at 4°C for 2–4 h, followed by fixation with 1% osmic acid in 0.1 M phosphate buffer (PB; pH 7.4) at 20°C for 2 h and dehydration with a graded alcohol series (50, 70, 80, 90, 95, 100, and 100%, every 15 min). Finally, the samples were infiltrated with a 1 : 1 mixture of acetone and SPI-Pon 812 overnight and embedded in SPI-Pon 812 epoxy resin overnight. Ultrathin sections (60–80 nm) were stained with 2% uranyl acetate, followed by lead citrate for 15 min each, and observed by transmission electron microscopy (TEM; Tecnai G^2^ F20 S-TWIN; FEI, Hillsboro, OR, USA).

### 2.8. Bacterial DNA Sequencing

Following homogenization of the mouse cecum and colon contents using glass beads in a Precellys 24 homogenizer (Bertin Technologies, Montigny, France), bacterial DNA was extracted using the QIAamp DNA Stool Mini Kit (Qiagen). Primers 319F (5′-ACTCCTACGGGAGGCAGCAG-3′) and 806R (5′-GGACTACHVGGGTWTCTAAT-3′) were used to amplify the V3-V4 domain of the 16S ribosomal RNA (rRNA) gene. PCR was performed as described previously [22]. The cycling parameters were as follows: 30 s of denaturation at 98°C, followed by 30 cycles of 15 s at 98°C, annealing for 15 s at 58°C, and elongation for 15 s at 72°C, with a final extension at 72°C for 60 s. The amplicons were then subjected to normalization, pooling, and pyrosequencing using the Illumina Miseq desktop sequencer (2 × 300 bp paired-end run).

QIIME (version 1.9.0, http://qiime.org) was used to perform sequence read processing, quality trimming, demultiplexing, and taxonomic assignments [23]. The alpha diversity, including the indexes of Shannon, Simpson, phylogenetic diversity- (PD-) whole tree, Chao1, and observed species, was calculated using QIIME. Weighted UniFrac principal coordinate analysis (PCoA) was performed using QIIME. Functional profiling of microbial communities was predicted by the phylogenetic investigation of communities by the reconstruction of unobserved states (PICRUSt) based on the Greengenes database [24]. The linear discriminant analysis (LDA) effect size (LEfSe) was conducted to estimate the effect size of each taxon with significant differential abundance [25]. The output file was further analyzed using statistical analysis of metagenomic profile (STAMP) software [26].

### 2.9. Statistical Analysis

We carried out two groups' comparisons by *t*-test (normal distribution and equal variance) or White's nonparametric *t*-test (nonnormal distribution) using SPSS (version 21.0; IBM Corp., Armonk, NY, USA). All the data are expressed as means ± standard deviation. A *p* value *<* 0.05 was deemed to indicate statistical significance.

## 3. Results

### 3.1. Overall Structural Changes of Microbial Communities following CCl_4_ and MSC Treatment

**Figure 1:**
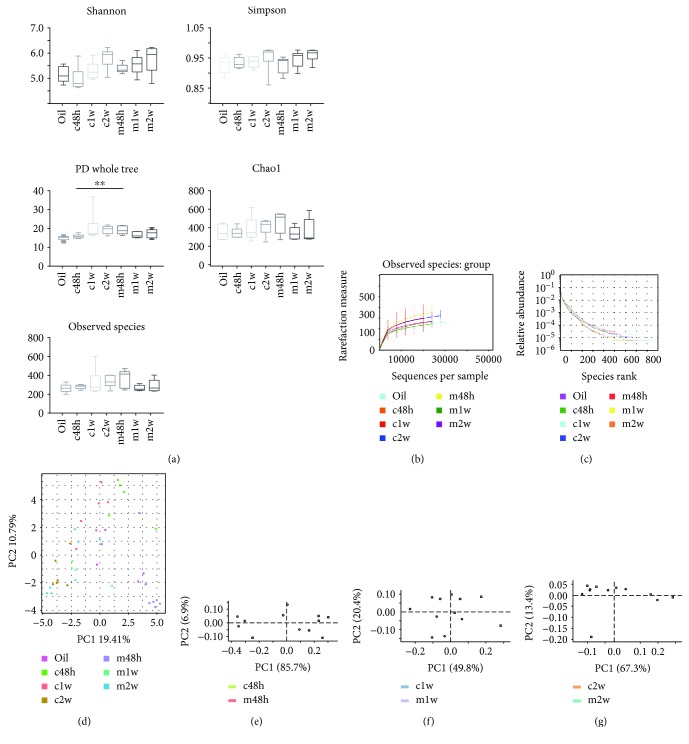
Comparison of the intestinal microbiota structure between the carbon tetrachloride- (CCl_4_-) treated and mesenchymal stem cell- (MSC-) transplanted groups. The cecum and colon contents were collected from C57BL/6 mice at 48 h, 1 w, and 2 w after performing CCl_4_ administration. (a) Alpha diversity measures (Shannon and Simpson indices), phylogenetic diversity whole-tree index, and richness indices (Chao1 and observed species). *n* = 6 per group; mean ± standard deviation (SD); *t*-test (^∗∗^ *p* < 0.01 vs. CCl_4_). (b) Rarefaction curves. (c) Rank abundance curves. (d) Principal coordinates analysis plot based on the UniFrac distance. Principal component analysis at 48 h (e), 1 w (f), and 2 w (g) using STAMP software. Oil: olive oil control; c: CCl_4_-treated group; m: MSC-transplanted group. 48 h, 1 w, and 2 w indicate 48 hours, 1 week, and 2 weeks after CCl_4_ treatment, respectively.

After the generation of multiplexed reads based on the nucleotide barcode of each sample and filtering the sequence reads for quality using QIIME, 2,132,586 high-quality sequences were acquired from all samples, with an average of 50,776 (range: 30,927–70,878) sequences per sample used for downstream statistical analysis. Specifically, 319,780 sequences were obtained from the olive oil control; 291,936, 314,004, and 313,643 sequences were obtained from the CCl_4_-treated groups (48 h, 1 w, and 2 w after CCl_4_ treatment, respectively); and 347,219, 273,154, and 272,850 sequences were obtained from the MSC-transplanted groups (48 h, 1 w, and 2 w after CCl_4_ treatment, respectively). All the sequences were clustered into 1517 operational taxonomic units (OTUs) using QIIME based on 97% sequence similarity and classified into 249 bacterial groups at the genus level. Each sample and group OTU number were calculated using QIIME. Specifically, there were 586 species-level OTUs in the olive oil control; 656, 896, and 794 OTUs in the CCl_4_-treated groups (48 h, 1 w, and 2 w after CCl_4_ treatment, respectively); and 713, 596, and 668 OTUs in the MSC-transplanted groups (48 h, 1 w, and 2 w after CCl_4_ treatment, respectively). Good coverage of all samples and groups was more than 99.5%, indicating sufficient community coverage. Details are shown in Table 1 and Table S1. At 48 h, compared with the CCl_4_-treated group, the MSC-transplanted group had a higher Shannon index (4.9732 vs. 5.3811) and lower Simpson index (0.9336 vs. 0.9279), and there were no statistically significant differences (*p* = 0.055 and *p* = 0.680, respectively). However, there were significant differences (*p* = 0.009) between the two groups at 48 h for the PD whole-tree index (15.8604 vs. 19.0074). The richness indices Chao1 (347.88065 vs. 459.8725) and observed species (280 vs. 374) between the two groups at 48 h showed no significant differences (*p* = 0.067 and *p* = 0.055). At 1 and 2 w, there were no significant differences between the CCl_4_-treated and MSC-transplanted groups for alpha diversity measures (Shannon and Simpson indices), PD whole-tree index, and richness indices Chao1 and observed species (Figure 1(a)).

Interestingly, the CCl_4_-treated groups showed steadily increased alpha diversity values (Shannon and Simpson indices) and richness indices (Chao1 and observed species) over time. The MSC-transplanted groups showed increased alpha diversity values (Shannon and Simpson indices) but decreased richness indices (Chao1 and observed species) over time (Table 1). Rarefaction curves for the observed species approached a plateau, indicating that the sequencing effort was sufficient in all samples for coverage of all OTUs (Figure 1(b), Supplementary Figure S2 online). Rank abundance curves fell slowly, indicating that samples were not dominated by a few OTUs but mostly low-abundance OTUs (Figure 1(c)). Alterations in the microbiota composition of all groups and samples were noted according to PCoA (Figure 1(d)). Additionally, principal component analysis (PCA) using STAMP software revealed that most of the samples from the CCl_4_-treated and MSC-transplanted groups were separated at 48 h and clustered together gradually from 1 to 2 w (Figures 1(e)–1(g)).

### 3.2. Alterations in the Intestinal Microbiota in Response to Acute Liver Injury and Administration of MSCs

**Figure 2:**
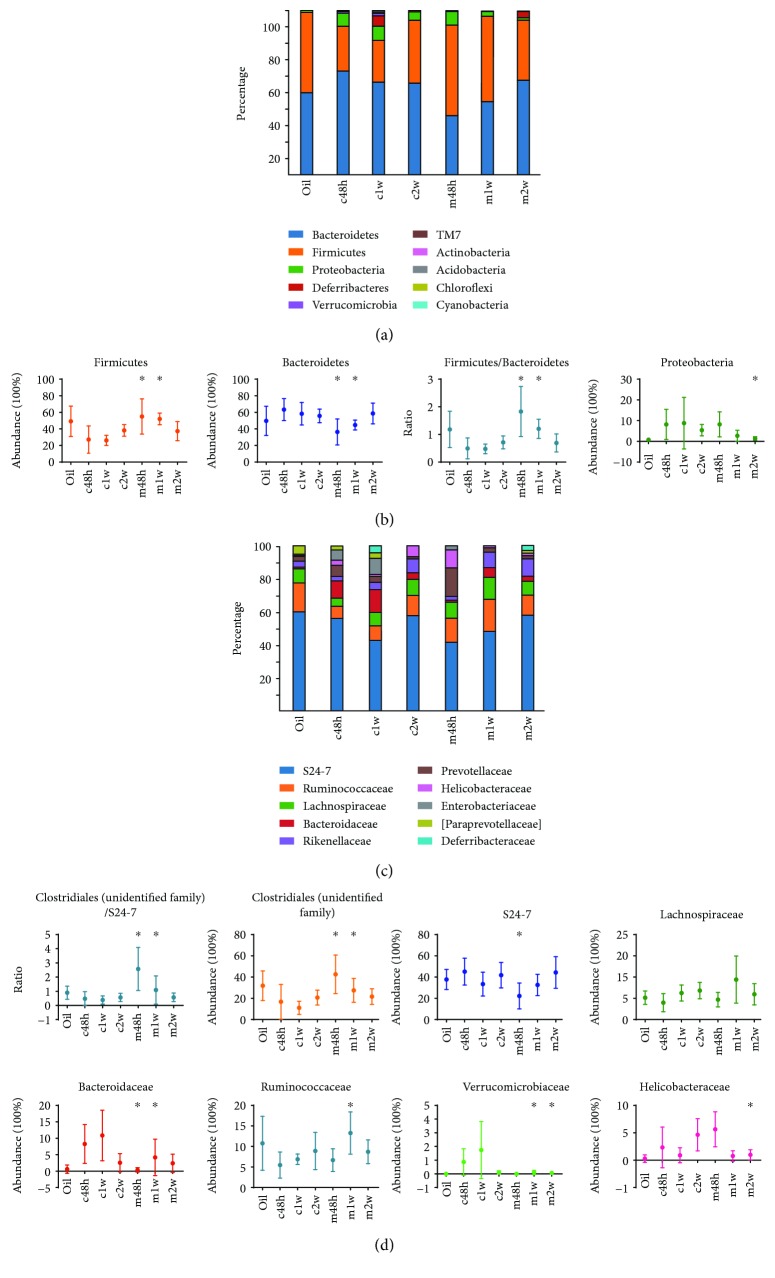
Microbial communities are changed following MSC treatment. Stacked bar charts (a) and point plots (b) representing changes in Firmicutes, Bacteroidetes, Proteobacteria, and the ratio of Firmicutes/Bacteroidetes over time. *n* = 6 per group; mean ± SD; White's nonparametric *t*-test (^∗^ *p* < 0.05 vs. CCl_4_). (c, d) Most abundant taxon changes over time at the family level. *n* = 6 per group; mean ± SD; White's nonparametric *t*-test (^∗^ *p* < 0.05 vs. CCl_4_). Oil: olive oil control; c: CCl_4_-treated group; m: MSC-transplanted group. 48 h, 1 w, and 2 w indicate 48 hours, 1 week, and 2 weeks after CCl_4_ treatment, respectively.

To characterize changes in the intestinal microbiota associated with CCl_4_-induced acute liver injury and administration of MSCs, we established and sequenced 16S rRNA amplicon libraries from the contents of the cecum and colon. Mice receiving an equal volume of olive oil followed by sacrifice at 48 h were used as controls. At the phylum level, the proportion of the five dominant bacterial phyla (Bacteroidetes, Firmicutes, Proteobacteria, Deferribacteres, and Verrucomicrobia) accounted for >95% of all sequences in all the groups (Figure 2(a)). Interestingly, the groups treated with CCl_4_ showed a steady increase in Firmicutes abundance but a reciprocal decrease in Bacteroidetes over time. The groups intravenously injected with MSCs exhibited opposite results, exhibiting a gradual increase in Bacteroidetes abundance but an inverse decrease in Firmicutes and Proteobacteria over time.

Furthermore, the ratios of Firmicutes/Bacteroidetes in the MSC-transplanted groups were decreased over time (Figure 2(b)). At the lower family level, *Clostridiales* (unidentified family), *Lachnospiraceae*, and *Ruminococcaceae* were the most abundant representatives of the Firmicutes phylum. The families *S24-7*, *Bacteroidaceae*, *Rikenellaceae*, *Prevotellaceae*, *Bacteroidales* (unidentified family), and *Paraprevotellaceae* were the most abundant representatives of the *Bacteroidetes* phylum (Figure 2(c)). The families *Enterobacteriaceae* and *Verrucomicrobiaceae* were the most abundant representatives of the Proteobacteria and Verrucomicrobia phyla, respectively. Overall, the MSC-treated group, compared with the CCl_4_-treated group, displayed *Clostridiales* (unidentified family) (16.8% to 42.6%, *p* = 0.036) expansion and *S24-7* (45.2% to 22.2%, *p* < 0.01), *Bacteroidaceae* (8.3% to 0.4%, *p* < 0.01), and *Verrucomicrobiaceae* (0.9% to 0.003%, *p* = 0.027) contraction at 48 h (Figure 2(d)). In the liver repair phase (1 w after CCl_4_ injection), the MSC-treated group, compared with the CCl_4_-treated group, showed *Clostridiales* (unidentified family) (11.0% to 27.5%, *p* < 0.01) and *Ruminococcaceae* (6.9% to 13.3%, *p* < 0.01) enrichment and decreased *Bacteroidaceae* (10.9% to 4.2%, *p* = 0.033) and *Verrucomicrobiaceae* (1.7% to 0.1%, *p* = 0.030). There were no differences between the MSC-treated and CCl_4_-treated groups 2 w after CCl_4_ injection except a relatively higher proportion of *Helicobacteraceae* (4.6% to 1.0%, *p* < 0.01) in the CCl_4_-treated group (Figure 2(d)). Accordingly, the ratios of *Clostridiales* (unidentified family)/*S24-7* in the MSC-transplanted groups were also decreased over time. Details of the statistical analysis at the phylum and family levels are shown in Supplemental Figure S3 online.

**Figure 3:**
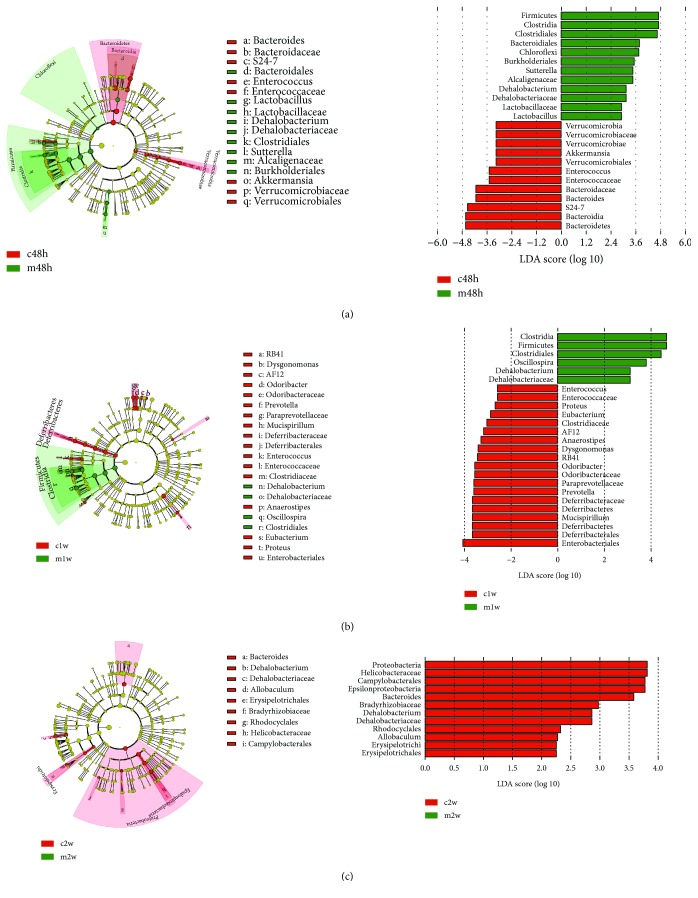
LDA for microbial community variation. Linear discriminative analysis (LDA) effect size (LEfSe; LDA score > 2) between the CCl_4_-treated and MSC-transplanted groups at 48 h (a), 1 w (b), and 2 w (c). At 2 w (c), there were no characteristic taxa in the MSC-transplanted group. c: CCl_4_-treated group; m: MSC-transplanted group. 48 h, 1 w, and 2 w indicate 48 hours, 1 week, and 2 weeks after CCl_4_ treatment, respectively.

To characterize the specific bacterial taxa at 48 h, 1 w, and 2 w after CCl_4_ treatment and MSC transplantation, the LEfSe algorithm was used. The cladograms that displayed the predominant bacterial taxa in the CCl_4_-treated and MSC-transplanted groups at different times are shown in Figure 3. The LEfSe analysis separately discovered 24, 25, and 12 discriminative features (LDA score > 2). The MSC-transplanted groups at 48 h and 1 w mainly showed the members of bacterial taxa in the Firmicutes phylum; those in the Bacteroidetes phylum were enriched in the CCl_4_-treated group at 48 h, whereas Verrucomicrobia, Deferribacteres, and Proteobacteria were enriched in the CCl_4_-treated groups at 48 h, 1 w, and 2 w, respectively (Figure 3), findings that were consistent with the above results. Interestingly, we found that the predominant families or genera did not always have high LDA scores, suggesting that the superimposed effect of some nonpredominant microflora may not be overlooked. At 48 h, *Sutterella*, *Dehalobacterium*, and *Lactobacillus* were most abundant in the MSC-transplanted group, and *f_S24-7*, *Bacteroides*, *Enterococcus*, and *Akkermansia* were most abundant in the CCl_4_-treated group. At 1 w, *Oscillospira* and *Dehalobacterium* were most abundant in the MSC-transplanted group, and *Proteus*, *Mucispirillum*, *Prevotella*, *Odoribacter*, *Dysgonomonas*, *AF12*, *Anaerostipes*, *Eubacterium*, and *Enterococcus* were most abundant in the CCl_4_-treated group. At 2 w, *f_Helicobacteraceae*, *f_Bradyhizobiaceae*, *Dehalobacterium*, and *Allobacullum* were most abundant only in the CCl_4_-treated group. In addition, the microbiota difference within different stages (48 h, 1 w, and 2 w) of the MSC-transplanted groups was also analyzed (48 h vs. 1 w; 48 h vs. 2 w). Some bacteria (*Prevotella*, *Flexispira*, *Holdemania*, *Ruminococcus*, *Sutterella*, *Anaerostipes*, *Eubacterium*, *Dysgonomonas*, *and Coprobacillus*) with high LDA scores were found in the MSC-transplanted group at 48 h. However, only *o_Burkholderiales*, *f_Alcaligenaceae*, and *g_Sutterella* were significantly changed in the MSC-transplanted group at 48 h, compared with the CCl_4_-treated group, suggesting that *Sutterella* may be more important to the MSC-transplanted group in the above mentioned bacteria.

### 3.3. Potential Functions of the Gut Microbiome in the CCl_4_-Treated and MSC-Transplanted Groups

**Figure 4:**
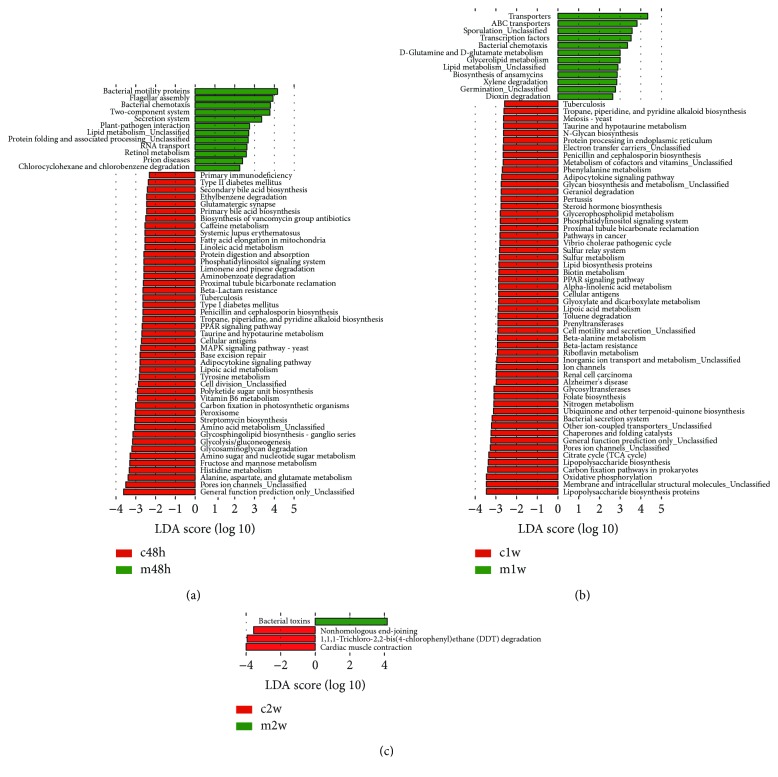
Kyoto Encyclopedia of Genes and Genomes (KEGG) pathway analysis. Statistically significant KEGG pathways between the CCl_4_-treated and MSC-transplanted groups were determined by STAMP software (White's nonparametric *t*-test), and LEfSe (LDA score > 2) of significant KEGG pathways was performed at 48 h (a), 1 w (b), and 2 w (c). c: CCl_4_-treated group; m: MSC-transplanted group. 48 h, 1 w, and 2 w indicate 48 hours, 1 week, and 2 weeks after CCl_4_ treatment, respectively. *n* = 6 per group.

Kyoto Encyclopedia of Genes and Genomes (KEGG; http://www.genome.jp/kegg/) pathways were predicted by PICRUSt based on the Greengenes database [24]. In addition, LEfSe was applied to study the effect size of each KEGG pathway with significant differential abundance.  Figure 4 shows 44, 54, and 3 biomarkers found at pathway level_3 in the CCl_4_-treated groups, and 12, 12, and 1 in the MSC-transplanted groups, at 48 h, 1 w, and 2 w, respectively. At 48 h, 27 metabolic pathways were found in the CCl_4_-treated group (61.4%; 27 of 44 pathways) under amino acid metabolism, carbohydrate metabolism, glycan biosynthesis and metabolism, biosynthesis of other secondary metabolites, energy metabolism, metabolism of cofactors and vitamins, metabolism of terpenoids and polyketides, metabolism of other amino acids, xenobiotic biodegradation, and metabolism and lipid metabolism categories.

In addition, the highest discriminating nonmetabolic pathway was “pore ion channels” under the cellular processes and signaling category. Furthermore, “cell division” was also noted. However, in the MSC-transplanted group, the three highest discriminatory powers of pathways were “bacterial motility proteins,” “flagellar assembly,” and “bacterial chemotaxis” under the cell motility category, followed by signal transduction and membrane transport categories. Three metabolic pathways were detected in the MSC-transplanted group under lipid metabolism, metabolism of cofactors and vitamins, xenobiotic biodegradation, and metabolism categories (Figure 4(a)). At 1 w, the pathway with the highest discriminatory power in the CCl_4_-treated group was the “lipopolysaccharide biosynthesis proteins” under the glycan biosynthesis and metabolism category. In addition, “lipopolysaccharide biosynthesis,” “glycosyltransferases,” and “N-glycan biosynthesis” were noted. The second discriminating pathway was “membrane and intracellular structural molecules” under the cellular processes and signaling category that also contains “pore ion channels,” “other ion-coupled transporters,” “inorganic ion transport and metabolism,” “cell motility and secretion,” and “electron transfer carrier” pathways in this group. Again, pathways in this group were mainly classified under the metabolism category (55.6%; 30 of 54 pathways). In the MSC-transplanted group, the two highest discriminatory powers of pathways were “transporters” and “ABC transporters” under the membrane transport category, followed by “sporulation (unclassified),” “transcription factors,” and “bacterial chemotaxis” pathways under the sporulation, transcription, and cell motility categories, respectively. Six metabolic pathways were detected in the MSC-transplanted group under the metabolism of other amino acids, lipid metabolism, metabolism of terpenoids and polyketides, xenobiotic biodegradation, and metabolism categories (Figure 4(b)). At 2 w, three pathways were found in the CCl_4_-treated group, and only one was detected in the MSC-transplanted group, indicating that the two groups tended to be consistent (Figure 4(c)).

### 3.4. Changes in the Intestinal Microbial Communities upon MSC Transplantation Improve Intestinal Histopathology and Epithelial Tight Junctions

**Figure 5:**
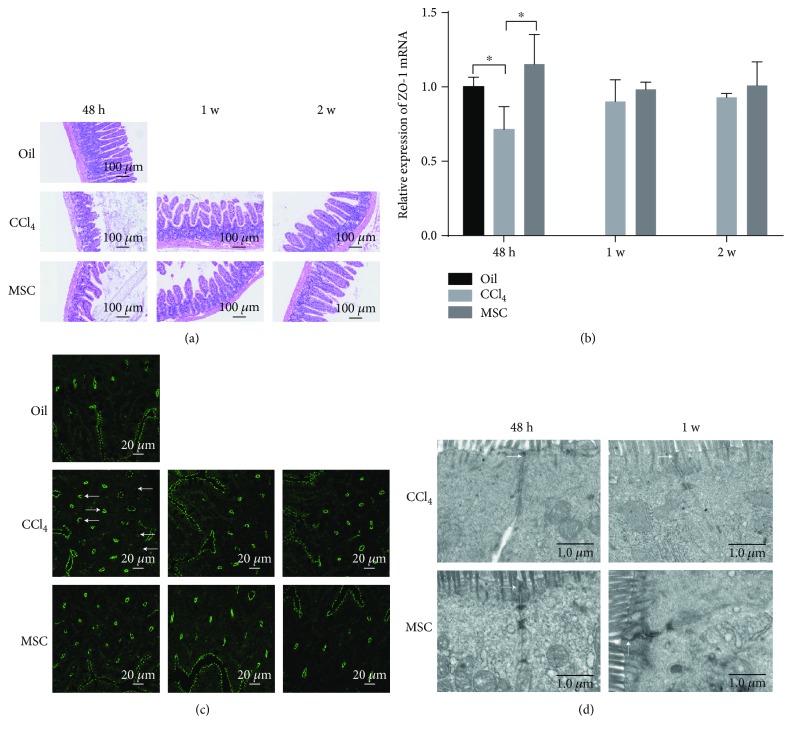
MSC transplantation improves intestinal histopathology and epithelial tight junctions (TJs). (a) Histopathology of the ileum of different experimental groups. (b) Relative expression of zonula occludens- (ZO-) 1 mRNA by real-time quantitative polymerase chain reaction. *n* = 4 per group; mean ± SD; *t*-test (^∗^ *p* < 0.05). (c) Immunofluorescence analysis of ZO-1 distribution. (d) Ultrastructure of TJs using transmission electron microscopy. Oil: olive oil control; c: CCl_4_-treated group; m: MSC-transplanted group. 48 h, 1 w, and 2 w indicate 48 hours, 1 week, and 2 weeks after CCl_4_ treatment, respectively.

The ileum histopathological findings from different experimental groups are shown in Figure 5(a). In the olive oil control mice, the ileum tissue exhibited normal villi, submucosa, inner and outer muscularis layers, and serosa. At 48 h, the ileum of CCl_4_-treated mice showed atrophy, erosions and sloughing of villi, decreased number of goblet cells, inner muscularis layer damage, and inflammatory cell infiltration. The ileum of mice transplanted with MSCs demonstrated scattered and slight shrinking of villi but regular architecture of the muscularis layers. At 1 w, the ileum of CCl_4_-treated mice displayed hyperplasia and disorderly arrangement of villi, while MSC-transplanted mice exhibited a normal arrangement and distribution of villi. At 2 w, the ileum of CCl_4_-treated and MSC-transplanted mice showed reversion to the normal length and arrangement of villi.

To evaluate epithelial tight junctions **(**TJs), we examined the expression and distribution of ZO-1, a protein that interacts directly with transmembrane TJ proteins [27], by qPCR and immunofluorescence analysis. At 48 h, MSC transplantation increased ZO-1 mRNA (m vs. c; *p* = 0.013; *t*-test) in the ileum but decreased ZO-1 mRNA (c vs. o, *p* = 0.029, *t*-test) in the CCl_4_-treated group. At 1 and 2 w, there were no significant differences between these groups, although ZO-1 expression was lower in the CCl_4_-treated groups (Figure 5(b)). Accordingly, immunofluorescence performed on ileal sections displayed discontinuous staining for ZO-1 at the apical cellular border [28, 29] at 48 h in the CCl_4_-treated group, suggesting a destroyed network of TJ proteins. Additionally, at 48 h, MSC transplantation improved epithelial TJs. At 1 and 2 w, both the CCl_4_-treated and MSC-transplanted groups showed intact epithelial TJs (Figure 5(c)). Because the TJ is a multiprotein complex, TEM was used in ultrathin sections to validate the ZO-1 results. The lower electron density of TJs was only evident in the CCl_4_-treated group at 48 h (Figure 5(d)).

### 3.5. MSCs Protect against CCl_4_-Induced Acute Liver Injury

**Figure 6:**
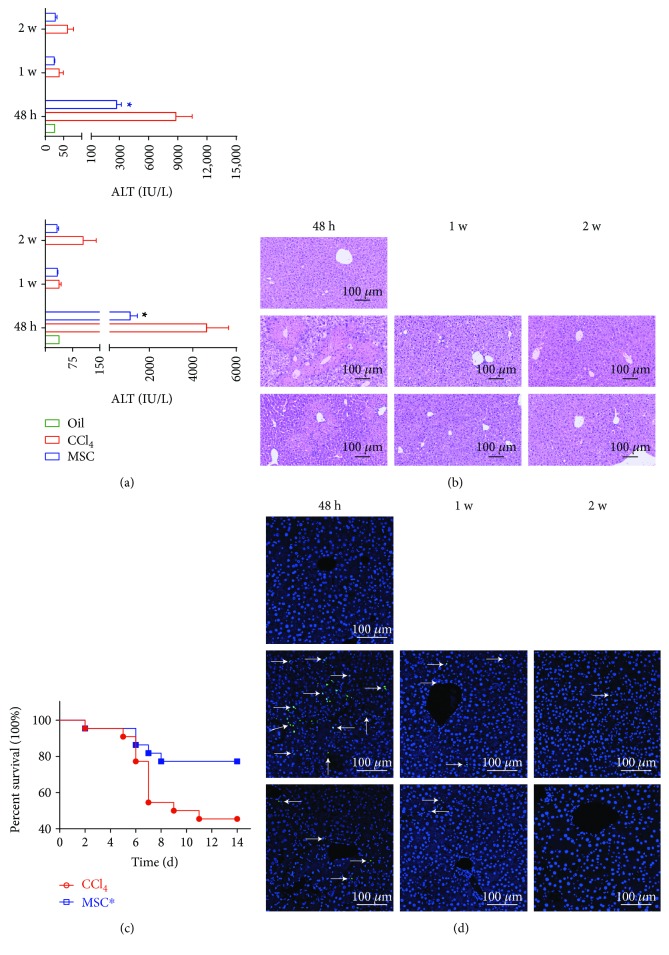
MSCs protect against CCl_4_-induced acute liver injury. (a) Liver alanine aminotransferase and aspartate aminotransferase levels. *n* = 4 per group; mean ± SD; *t*-test (^∗^ *p* < 0.05 vs. CCl_4_). (b) Hematoxylin and eosin staining of mouse livers. (c) Kaplan–Meier plots for mouse survival after injection of CCl_4_ (1.5 mL/kg), followed by MSC administration 6 h later. *n* = 22 mice for each bar (^∗^ *p* < 0.05 vs. CCl_4_). (d) TUNEL staining of liver sections and DAPI were applied for nucleus staining, representative of images with similar results. Oil: olive oil control; c: CCl_4_-treated group; m: MSC-transplanted group. 48 h, 1 w, and 2 w indicate 48 hours, 1 week, and 2 weeks after CCl_4_ treatment, respectively.

Mouse ALT and AST levels, hepatic histopathology, and survival curves were assayed to evaluate the therapeutic effect of MSCs. The results showed elevated serum levels of ALT and AST and widespread areas of hepatocellular necrosis and steatosis at 48 h in the CCl_4_-treated group, but significantly reduced serum levels of ALT and AST and the total size of necrosis and steatosis areas in the MSC transplantation group (Figures 6(a) and 6(b)). At 1 w, the CCl_4_-treated group showed normal biochemistry but punctate necrosis, while the MSC-transplanted group showed biochemical and histological recovery. At 2 w, both the CCl_4_-treated and MSC-transplanted groups showed normal biochemistry and liver histology. Furthermore, MSC transplantation ameliorated mouse survival significantly from 45.5% to 77.3% at 2 w (Figure 6(c)) and reduced apoptosis at 48 h (Figure 6(d)).

## 4. Discussion

Our study systematically analyzed gut microbial changes associated with MSC therapy for CCl_4_-induced acute liver injury. Functional KEGG pathway analysis demonstrated specific and distinct pathways of intestinal microbiota at designated times (48 h, 1 w, and 2 w) associated with liver injury and repair. Moreover, based on microbiota changes, ileum histopathology and epithelial TJs were assessed. Furthermore, the therapeutic effect of MSCs on CCl_4_-induced acute liver injury was reevaluated.

The effects of CCl_4_ administration on liver cell injury have largely been studied in mice and, although the effects appear to be related to genetic strain determinants, C57BL/6 mice showed intermediate lesions between the more susceptible BALB/c strain, which shows histological recovery by 3 w, and the less susceptible SJL/J strain, which shows histological recovery by 1 w [30]. Accordingly, in the presented data, C57BL/6 mice exhibited normal biochemistry and liver histology 2 w after i.p. injection with 3 mL/kg 50% (*v*/*v*) CCl_4_. Moreover, transplantation with MSCs showed a significant therapeutic effect, with biochemical and histological recovery at 1 w. Although CCl_4_ intoxication does not result in permanent liver damage, manifested as massive necrosis at 48 h followed by a period of repair [31], 66.7% (8/12) of mice died on day 6 or 7, indicating that other factors lead to sustained hepatic damage or death. Because the gut-liver axis plays a key role in hepatic disease [32], we assumed that gut microbiota also plays an important role in the outcome of this model. Indeed, the liver with transplantation of 5 × 10^5^ MSCs showed a considerable effect on the composition of the microbial communities compared with no transplantation, despite no significant differences in the alpha diversity measurements except the PD whole-tree index between the groups, possibly because of the small sample sizes of both groups.

At 48 h and 1 w, higher Firmicutes abundance was linked to *Clostridiales* (unidentified family) and *Ruminococcaceae*, while Bacteroidetes contraction was linked to decreased *S24-7* and *Bacteroidaceae* in the MSC-transplanted group. Firmicutes and Bacteroidetes are the most abundant bacterial phyla affecting host physiology in both humans and mice [33, 34]. An imbalanced Firmicutes/Bacteroidetes ratio has been associated with various disease processes [35]. For instance, a high-fat diet and ob/ob mice have increased Firmicutes, and ob/ob mice also have lower Bacteroidetes over time compared with lean controls [36, 37]. In addition, obese individuals have fewer Bacteroidetes and more Firmicutes than lean controls [38], and reduced Bacteroidetes, specifically the families *S24-7* and *Bacteroidaceae*, were detected during the immune-priming phase of arthritis [39]. However, NASH patients have decreased abundance of the families *Lachanospiraceae* and *Ruminococcaceae*, which belong to the phylum Firmicutes, and increased abundance of the family *Prevotellaceae* and *Enterobacteriaceae*, which belong to the phylum Bacteroidetes and Proteobacteria, respectively [40]. In addition, diabetic mice have a lower Firmicutes/Bacteroidetes ratio [41], and a subset of Crohn's disease and ulcerative colitis patients is characterized by depletion of Firmicutes and Bacteroidetes with the relative expansion of Proteobacteria [42]. At 2 w, the most obvious difference in the gut microbiome between the CCl_4_-treated and MSC-transplanted groups was a relatively high proportion of the family *Helicobacteraceae*, which is generally associated with gastrointestinal tract diseases of animals belonging to the phylum Proteobacteria [43]. In addition, there was almost no evidence of the family *Verrucomicrobiaceae*, which only includes the genus *Akkermansia*, in the MSC-transplanted and olive oil control groups. A previous report revealed that the mean relative abundance values of *Akkermansia* in the gut microbiota of mice and healthy human adults are 0.003% and 0.744%, respectively [44]. The mucus-degrading bacteria *Akkermansia*, which initiate mucus degradation to produce oligosaccharides and acetate, resulting in colonized bacterial growth and resistance to pathogenic bacteria, are beneficial for mucus-associated microbiota composition [45]. Moreover, the mucus-colonizing microbes have been considered to contribute to the restoration of the microbiota [46]; *in vitro*, *Akkermansia* can adhere to the intestinal epithelium and strengthen enterocyte monolayer integrity [47]. Reactive growth of this bacterium may be one of the reasons for gut microflora self-recovery of the CCl_4_-treated groups. Interestingly, our data also demonstrated that the predominant families or genera do not always have high LDA scores. At 48 h and 1 w, *Sutterella*, *Dehalobacterium*, *Lactobacillus* (species *reuteri*), and *Oscillospira* were the most abundant genera in the MSC-transplanted groups. *Lactobacillus reuteri* can significantly increase the expression of TJ-associated proteins [48] and decrease bacterial translocation [49], as well as reduce serum triglycerides and increase the ratio of high-density lipoprotein to low-density lipoprotein [50]. It also has potent direct anti-inflammatory effects on epithelial cells by upregulating the anti-inflammatory molecule nerve growth factor and inhibiting nuclear factor *κ*B (NF-*κ*B) translocation to the nucleus [51]. It has been shown that *Dehalobacterium* and *Oscillospira* can prevent atherosclerosis, possibly through lipid metabolism [52]. However, little is known about the role of unclassified *Sutterella* in the intestinal tract.

The gut microflora, as a forgotten organ, has collective metabolic and immunoregulation abilities that are relevant to host health and disease [53]. The potential functions of the gut microbiome in the MSC-transplanted groups differed significantly during liver injury and the repair phase, such as at 48 h and 1 w, compared with those in the CCl_4_-treated groups. At 48 h and 1 w, the microbiota in MSC-transplanted mice revealed biomarkers involved in cell motility, signal transduction, membrane transport, transcription, and metabolism of lipid, cofactors, vitamins, terpenoids, and polyketides, as well as xenobiotics, which are beneficial for maintaining the normal hepatic condition [35]. Especially at 48 h (liver injury phase), the MSC-transplanted group significantly enriched the functional genes involved in cell motility (“bacterial motility proteins,” “flagellar assembly,” and “bacterial chemotaxis”). This suggests that certain bacterial movements may be beneficial for adaptation and response to stimuli (such as changes in bile acids and/or systemic inflammatory factors). Because the above three pathways were significantly correlated with the ratio of Firmicutes/Bacteroidetes at 48 h (*r* > 0.9; *p* < 0.05; Spearman correlation analysis), it is possible that bacterial movements facilitate the growth and proliferation of some Firmicutes microorganisms, inhibit the proliferation of LPS-producing bacteria, and maintain the barrier integrity and homeostasis of the intestinal tract. Reducing the transfer of intestinal bacterial potentially proinflammatory products to the liver may be beneficial for liver repair. Surprisingly, at 48 h and 1 w, the highest discriminating nonmetabolic pathway of the gut microbiome in the CCl_4_-treated groups was the cellular processes and signaling category, followed by liver regeneration-associated gene expression [35], and the highest discriminating metabolic pathways, amino acid metabolism and glycan biosynthesis and metabolism, may also function in liver regeneration [35, 54].

## 5. Conclusion

In summary, our data further indicate the extensive role of MSCs in rescuing acute liver injury induced by CCl_4_. It is possible that initial alterations in the Firmicutes/Bacteroidetes ratio maintain intestinal mucosal biology and homeostasis, which benefit liver repair.

## Figures and Tables

**Table 1:** Number of sequences and operational taxonomic units, good coverage estimation, and diversity index for each group from the pyrosequencing analysis.

Group^1^	No. of reads	No. of OTUs^2^	Good coverage	Richness indices				Alpha diversity	
				Chao1	95% CI	Observed species	95% CI	Shannon	Simpson
Oil	319,780	586	99.78%	353	270.7–434.4	264	218–309	5.143298	0.928244
c48h	291,936	656	99.78%	348	291.3–404.3	280	256–304	4.973242	0.933629
c1w	314,004	896	99.77%	386	251.7-520.5	324	177-470	5.308093	0.936644
c2w	313,643	794	99.76%	409	319.0-498.2	335	269-400	5.811608	0.952789
m48h	347,219	713	99.70%	460	339.3-580.5	374	276-472	5.381068	0.927921
m1w	273,154	596	99.78%	342	269.2-415.4	265	233-296	5.546923	0.947188
m2w	272,850	668	99.78%	364	228.8-499.3	289	220-358	5.763545	0.960001

^1^Oil: olive oil control; c: carbon tetrachloride- (CCl_4_-) treated group; m: mesenchymal stem cell- (MSC-) transplanted group. 48 h, 1 w, and 2 w indicate 48 h, 1 week, and 2 weeks following CCl_4_ treatment, respectively. *n* = 6 per group. ^2^Sequences were clustered into operational taxonomic units (OTUs) based on 97% sequence similarity.

## Data Availability

All supporting data are included in the article and its additional files.

## Supplementary Materials

Supplementary Materials Figure S1: immunophenotypic and differentiation analysis of mouse compact bone-derived MSCs. (a) Fluorescence-activated cell sorting results showed that MSCs (passage 7) were positive for CD44 (96.30%), CD105 (53.33%), and CD29 (99.95%) and the progenitor cell marker Sca-1 (95.88%) but negative for CD45 (2.89%), CD34 (9.53%), and CD11b (2.11%); the endothelial cell marker CD31 (1.03%); and costimulating molecules Ia (0.62%) and CD86 (1.86%). (b) These cells showed classic spindle-shaped morphology. (c) Osteoblastogenesis of MSCs was assayed with Alizarin Red S on day 21. (d) Adipogenesis was assessed by staining with Oil Red O on day 28. Figure S2: Rarefaction curves for the observed species in the gut microbiota from olive oil controls (*n* = 6), CCl4-treated mice (*n* = 18), and MSC-transplanted mice (*n* = 18). Oil: olive oil control; c: CCl4-treated group; m: MSC-transplanted group. 48 h, 1 w, and 2 w indicate 48 hours, 1 week, and 2 weeks after CCl_4_ treatment. Figure S3. Statistical analysis of CCl_4_-treated mice (*n* = 6) and MSC-transplanted mice (*n* = 6) at the phylum and family levels at 48 h, 1 w, and 2 w, respectively. Significant differences were determined using White's nonparametric *t*-test with an average abundance of taxon > 1%, *p* < 0.05 and 95% confidence intervals. c: CCl_4_-treated group; m: MSC-transplanted group. 48 h, 1 w, and 2 w indicate 48 hours, 1 week, and 2 weeks after CCl_4_ treatment, respectively. Table S1: number of sequences and operational taxonomic units, good coverage estimation, and diversity index for each sample from the pyrosequencing analysis.
