# Supplementary material for: Characteristics of Intestinal Microecology during Mesenchymal Stem Cell-Based Therapy for Mouse Acute Liver Injury
Source: Stem Cells Int. 2019 Feb 5;2019:2403793. doi: 10.1155/2019/2403793 (PMC6379839; doi:10.1155/2019/2403793)
Supplement: Supplementary Materials — Figure S1: immunophenotypic and differentiation analysis of mouse compact bone-derived MSCs. (a) Fluorescence-activated cell sorting results showed that MSCs (passage 7) were positive for CD44 (96.30%), CD105 (53.33%), and CD29 (99.95%) and the progenitor cell marker Sca-1 (95.88%) but negative for CD45 (2.89%), CD34 (9.53%), and CD11b (2.11%); the endothelial cell marker CD31 (1.03%); and costimulating molecules Ia (0.62%) and CD86 (1.86%). (b) These cells showed classic spindle-shaped morphology. (c) Osteoblastogenesis of MSCs was assayed with Alizarin Red S on day 21. (d) Adipogenesis was assessed by staining with Oil Red O on day 28. Figure S2: Rarefaction curves for the observed species in the gut microbiota from olive oil controls (n = 6), CCl4-treated mice (n = 18), and MSC-transplanted mice (n = 18). Oil: olive oil control; c: CCl4-treated group; m: MSC-transplanted group. 48 h, 1 w, and 2 w indicate 48 hours, 1 week, and 2 weeks after CCl4 treatment. Figure S3. Statistical analysis of CCl4-treated mice (n = 6) and MSC-transplanted mice (n = 6) at the phylum and family levels at 48 h, 1 w, and 2 w, respectively. Significant differences were determined using White's nonparametric t-test with an average abundance of taxon > 1%, p < 0.05 and 95% confidence intervals. c: CCl4-treated group; m: MSC-transplanted group. 48 h, 1 w, and 2 w indicate 48 hours, 1 week, and 2 weeks after CCl4 treatment, respectively. Table S1: number of sequences and operational taxonomic units, good coverage estimation, and diversity index for each sample from the pyrosequencing analysis. [file 2403793.f1.docx]

**Supplemental Data**

Characteristics of intestinal microecology during mesenchymal stem cell-based therapy for mouse acute liver injury

Xiaotian Dong^1^, Xudong Feng^1^, Jingqi Liu^1^, Yanping Xu^1^, Qiaoling Pan^1,2^, Zongxin Ling^1,2^, Jiong Yu^1,2^, Jinfeng Yang^1,2^, Lanjuan Li^1,2^, Hongcui Cao*^1,2^

1 State Key Laboratory for the Diagnosis and Treatment of Infectious Diseases, First Affiliated Hospital, College of Medicine, Zhejiang University, 79 Qingchun Rd., Hangzhou City 310003, China

2 Collaborative Innovation Center for Diagnosis and Treatment of Infectious Diseases, 79 Qingchun Rd., Hangzhou City 310003, China

*Corresponding author:

Hongcui Cao, State Key Laboratory for the Diagnosis and Treatment of Infectious Diseases, First Affiliated Hospital, College of Medicine, Zhejiang University; Collaborative Innovation Center for the Diagnosis and Treatment of Infectious Diseases, 79 Qingchun Rd., Hangzhou City 310003, China. Tel: 86-571-87236451; Fax: 86-571-87236459

E-mail: [hccao@zju.edu.cn](mailto:hccao@zju.edu.cn)

**Figure S1. Immunophenotypic and differentiation analysis of mouse compact bone-derived MSCs.** (A) Fluorescence-activated cell sorting results showed that MSCs (passage 7) were positive for CD44 (96.30%), CD105 (53.33%), and CD29 (99.95%), and the progenitor cell marker Sca-1 (95.88%) but negative for CD45 (2.89%), CD34 (9.53%), and CD11b (2.11%), the endothelial cell marker CD31 (1.03%), and co-stimulating molecules Ia (0.62%) and CD86 (1.86%). (B) These cells showed classic spindle-shaped morphology. (C) Osteoblastogenesis of MSCs was assayed with Alizarin Red S on day 21. (D) Adipogenesis was assessed by staining with Oil Red O on day 28.


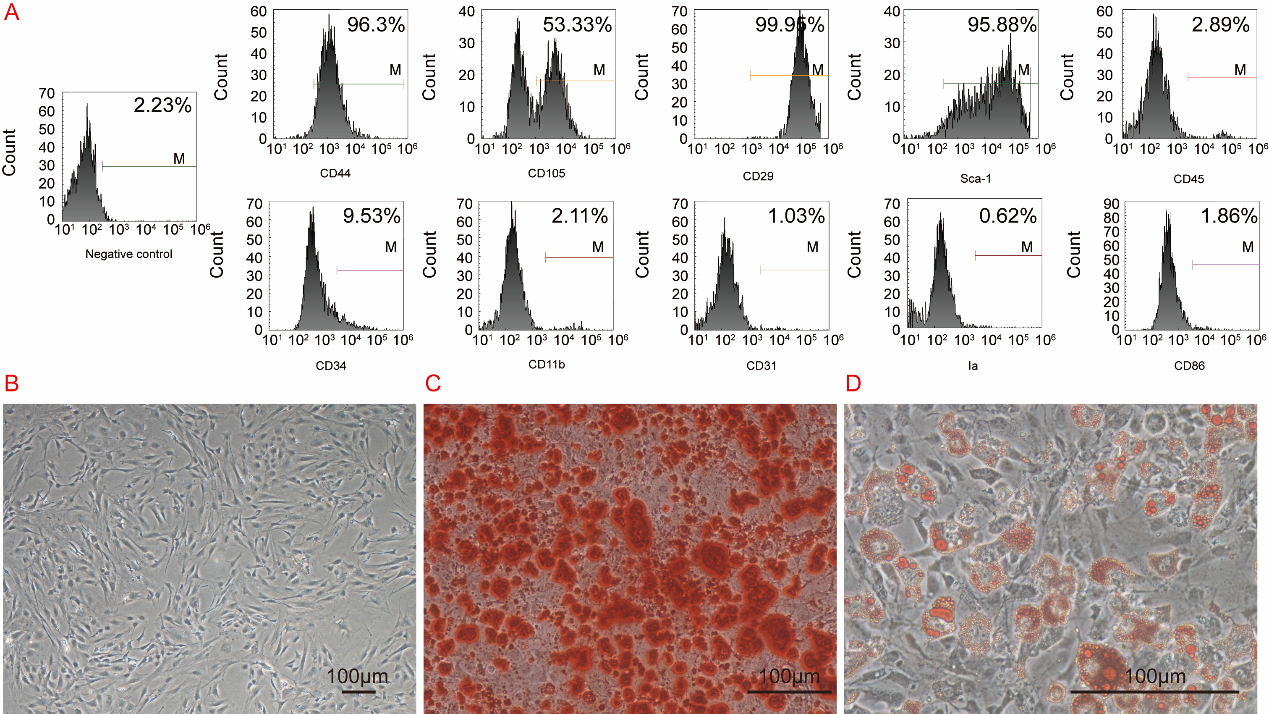


**Figure S2. Rarefaction curves for the observed species in the gut microbiota from olive oil controls (n = 6), CCl_4_-treated mice (n = 18), and MSC-transplanted mice (n = 18).** Oil: olive oil control; c: CCl_4_-treated group; m: MSC-transplanted group. 48, 1 w, and 2 w indicate 48 hours, 1 week, and 2 weeks after CCl_4_ treatment.


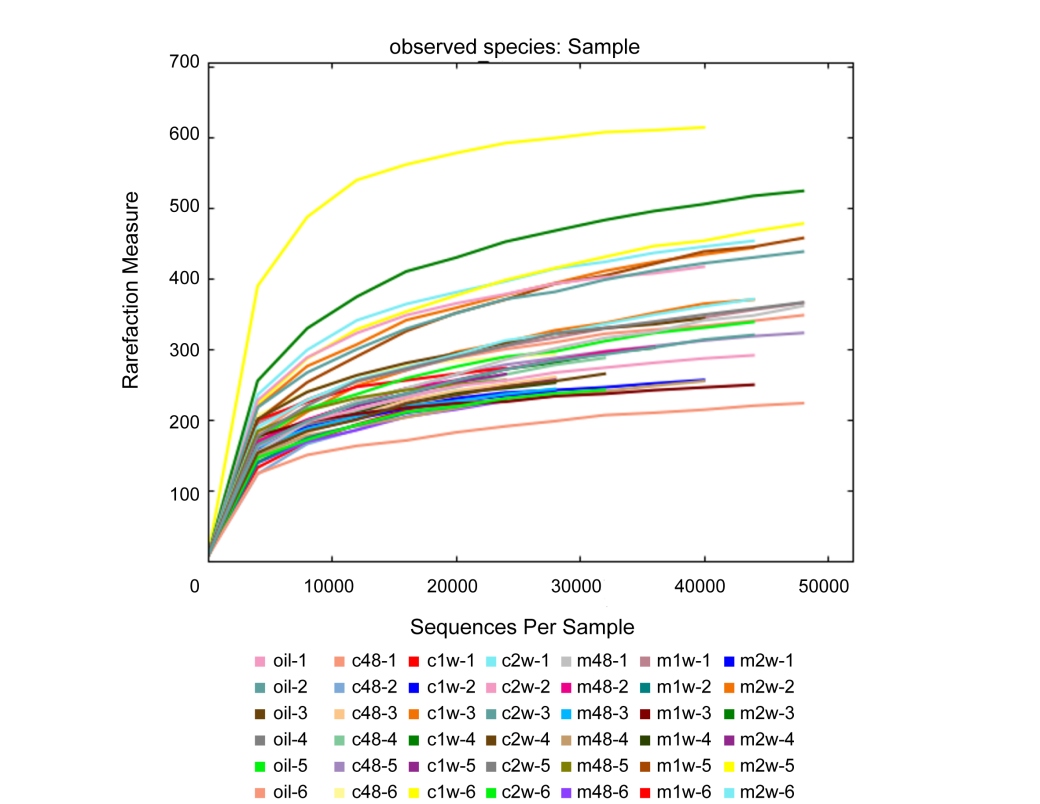


**Figure S3. Statistical analysis of CCl_4_-treated mice (n = 6) and MSC-transplanted mice (n = 6) at the phylum and family levels at 48 h, 1 w, and 2 w, respectively.** Significant differences were determined using White’s non-parametric *t*-test with an average abundance of taxon > 1%, *p* < 0.05, and 95% confidence intervals. c: CCl_4_-treated group; m: MSC-transplanted group. 48, 1 w, and 2 w indicate 48 hours, 1 week, and 2 weeks after CCl_4_ treatment, respectively.


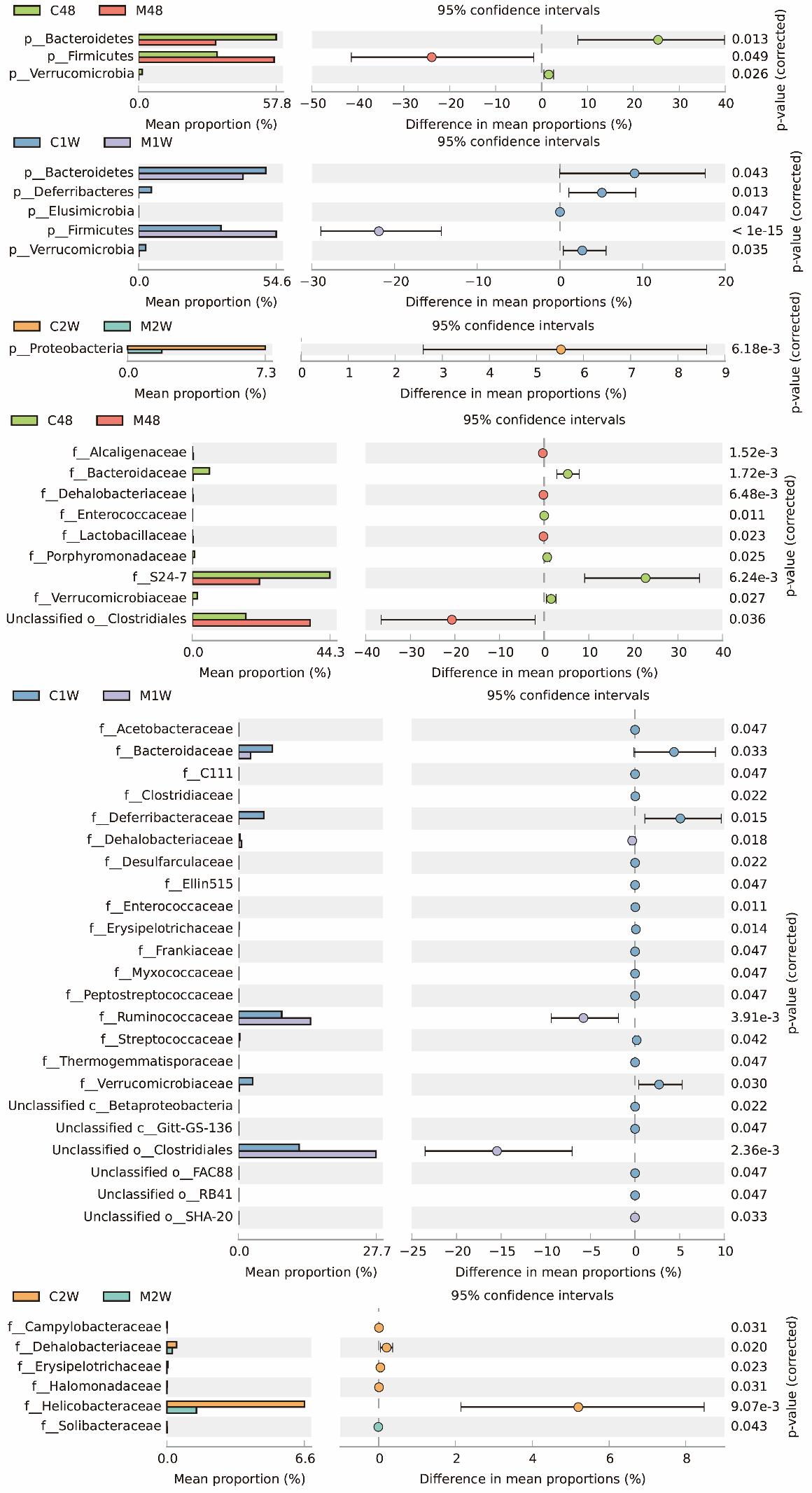


**Table S1. Number of sequences and operational taxonomic units, good coverage estimation, and diversity index for each sample from the pyrosequencing analysis.**

| Sample^1^ | Sample Size | OTUs Number | Goods coverage | Shannon | Simpson | PDwholetree | Chao1 | Observed species |
| --- | --- | --- | --- | --- | --- | --- | --- | --- |
| Oil-1 | 54484 | 294 | 0.998133 | 5.062282 | 0.937102 | 15.1256 | 315 | 268 |
| Oil-2 | 48524 | 326 | 0.997200 | 4.933261 | 0.907550 | 15.7618 | 452 | 286 |
| Oil-3 | 42943 | 272 | 0.997567 | 5.566840 | 0.962538 | 15.5259 | 360 | 259 |
| Oil-4 | 65717 | 372 | 0.997000 | 5.436499 | 0.946839 | 15.9826 | 437 | 329 |
| Oil-5 | 44788 | 247 | 0.998467 | 5.127200 | 0.932483 | 14.4362 | 272 | 239 |
| Oil-6 | 63324 | 225 | 0.998533 | 4.733707 | 0.882952 | 12.9319 | 279 | 200 |
| c48h-1 | 70081 | 357 | 0.997900 | 4.701068 | 0.914379 | 15.7764 | 368 | 305 |
| c48h-2 | 37920 | 243 | 0.997269 | 4.735798 | 0.934051 | 14.6273 | 297 | 243 |
| c48h-3 | 36785 | 263 | 0.997792 | 4.626073 | 0.919653 | 16.0567 | 335 | 263 |
| c48h-4 | 50969 | 297 | 0.997000 | 4.840146 | 0.922673 | 15.8812 | 442 | 282 |
| c48h-5 | 60837 | 325 | 0.997633 | 5.053669 | 0.948562 | 15.1154 | 346 | 291 |
| c48h-6 | 35344 | 294 | 0.999307 | 5.882697 | 0.962453 | 17.7052 | 299 | 294 |
| c1w-1 | 44667 | 228 | 0.996470 | 5.091416 | 0.949105 | 17.1991 | 330 | 228 |
| c1w-2 | 34606 | 241 | 0.998737 | 5.033597 | 0.929550 | 17.0275 | 258 | 241 |
| c1w-3 | 63104 | 379 | 0.996800 | 5.395714 | 0.953324 | 16.2163 | 440 | 326 |
| c1w-4 | 52512 | 305 | 0.997633 | 5.445426 | 0.955736 | 17.4699 | 360 | 287 |
| c1w-5 | 64380 | 260 | 0.997959 | 4.962010 | 0.922747 | 18.0717 | 312 | 260 |
| c1w-6 | 54735 | 618 | 0.998600 | 5.920397 | 0.909400 | 36.7509 | 617 | 600 |
| c2w-1 | 54216 | 375 | 0.996900 | 5.992124 | 0.971657 | 19.1297 | 437 | 330 |
| c2w-2 | 56424 | 420 | 0.997400 | 5.028964 | 0.860829 | 20.7487 | 462 | 402 |
| c2w-3 | 61394 | 440 | 0.996700 | 5.950953 | 0.968266 | 20.5174 | 481 | 400 |
| c2w-4 | 51490 | 346 | 0.997667 | 6.222506 | 0.976281 | 21.8421 | 388 | 330 |
| c2w-5 | 35464 | 234 | 0.999108 | 5.940038 | 0.973748 | 16.1290 | 247 | 234 |
| c2w-6 | 54655 | 339 | 0.996933 | 5.735062 | 0.965956 | 17.7224 | 438 | 311 |
| m48h-1 | 50205 | 258 | 0.998800 | 5.311016 | 0.942940 | 16.2367 | 271 | 245 |
| m48h-2 | 56544 | 449 | 0.996333 | 5.293582 | 0.906198 | 18.1992 | 515 | 402 |
| m48h-3 | 69551 | 538 | 0.996600 | 5.704724 | 0.941717 | 21.3042 | 549 | 471 |
| m48h-4 | 30927 | 271 | 0.997179 | 5.337087 | 0.941061 | 17.4338 | 364 | 271 |
| m48h-5 | 70878 | 497 | 0.996433 | 5.180758 | 0.882596 | 19.3347 | 514 | 427 |
| m48h-6 | 69114 | 459 | 0.996633 | 5.459243 | 0.953012 | 21.5359 | 545 | 428 |
| m1w-1 | 63676 | 369 | 0.996333 | 4.935120 | 0.898410 | 18.2946 | 448 | 312 |
| m1w-2 | 50319 | 313 | 0.997400 | 5.730421 | 0.957508 | 15.9388 | 386 | 289 |
| m1w-3 | 35810 | 245 | 0.998546 | 5.650070 | 0.958655 | 16.1051 | 281 | 245 |
| m1w-4 | 51057 | 259 | 0.998133 | 5.358215 | 0.931183 | 15.3269 | 296 | 237 |
| m1w-5 | 33567 | 261 | 0.999031 | 6.109017 | 0.976469 | 18.3508 | 275 | 261 |
| m1w-6 | 38725 | 244 | 0.997567 | 5.498696 | 0.960904 | 15.0535 | 368 | 243 |
| m2w-1 | 68294 | 376 | 0.997033 | 6.127174 | 0.973932 | 20.4890 | 456 | 330 |
| m2w-2 | 31414 | 233 | 0.998095 | 4.795498 | 0.918280 | 14.1049 | 277 | 233 |
| m2w-3 | 45180 | 250 | 0.998700 | 6.226477 | 0.976058 | 15.3316 | 292 | 239 |
| m2w-4 | 35417 | 255 | 0.998582 | 5.498013 | 0.956355 | 17.5997 | 284 | 255 |
| m2w-5 | 59519 | 458 | 0.995733 | 5.776189 | 0.961145 | 18.9145 | 587 | 402 |
| m2w-6 | 33026 | 276 | 0.998917 | 6.157920 | 0.974237 | 17.8418 | 289 | 276 |

^1^ Oil: olive oil control; c: carbon tetrachloride (CCl_4_)-treated group; m: mesenchymal stem cell (MSC)-transplanted group. 48h, 1 w, and 2 w indicate 48 hours, 1 week, and 2 weeks after CCl_4_ treatment, respectively.
